# A Steroidal Saponin from *Ophiopogon japonicus* Extends the Lifespan of Yeast via the Pathway Involved in *SOD* and *UTH1*

**DOI:** 10.3390/ijms14034461

**Published:** 2013-02-25

**Authors:** Kaiyue Sun, Shining Cao, Liang Pei, Akira Matsuura, Lan Xiang, Jianhua Qi

**Affiliations:** 1 College of Pharmaceutical Sciences, Zhejiang University, Yu Hang Tang Road 866, Hangzhou 310058, China; E-Mails: sunkaiyue21@163.com (K.S.); 21019004@zju.edu.cn (S.C.); qijianhua@zju.edu.cn (J.Q.); 2 College of Biology and Science, Sichuan Agricultural University, Yaan, Sichuan 625014, China; E-Mail: 839015@163.com; 3 Department of Nanobiology, Graduate School of Advanced Integration Science, Chiba University, Chiba 263-8522, Japan; E-Mail: amatsuur@faculty.chiba-u.jp

**Keywords:** nolinospiroside F, *SOD*, *UTH1*, anti-aging, anti-oxidative stress

## Abstract

Nolinospiroside F is a steroidal saponin isolated from *Ophiopogon japonicus* (*O. japonicus*). In this study, we found that nolinospiroside F significantly extends the replicative lifespan of K6001 yeast at doses of 1, 3 and 10 μM, indicating that it has an anti-aging effect. This may be attributed to its anti-oxidative effect, as nolinospiroside F could increase yeast survival under oxidative stress conditions and decrease the level of malondialdehyde (MDA), an oxidative stress biomarker. It could also increase anti-oxidative stress genes, *SOD1* and *SOD2*, expression, and the activity of superoxide dismutase (SOD). It increase the activity of SIRT1, an upstream inducer of *SOD2* expression. In *sod1* and *sod2* mutant yeast strains, nolinospiroside F failed to extend their replicative lifespan. These results indicate that SOD participates in the anti-aging effect of nolinospiroside F. Furthermore, nolinospiroside F inhibited the expression of *UTH1*, a yeast-aging gene that is involved in the oxidative stress of yeast, and failed to extend the replicative lifespan of *uth1* or *skn7* mutant yeast cells. SKN7 is the transcriptional activator of *UTH1*. We also demonstrate that *SOD* and *UTH1* regulate each other’s expression. Together, these results suggest that *SOD* and *UTH1* genes are required for and play interactive roles in nolinospiroside F-mediated yeast lifespan extension.

Abbreviations

AD: Alzheimer’s disease
MS: mass spectrometry
MDA: malondialdehyde
NMR: nuclear magnetic resonance
*O. japonicus*: *Ophiopogon japonicus*
RES: resveratrol
ROS: reactive oxygen species
RT-PCR: real-time polymerase chain reaction
SOD: superoxide dismutase
YPD: yeast peptone dextrose
YPGal: yeast peptone galactose

## 1. Introduction

Aging has become a worldwide problem, and increasing numbers of people are suffering from age-related diseases, such as Alzheimer’s disease (AD) and diabetes. Recent studies have shown a close relationship between aging and age-related disorders, especially between aging and AD. Small molecules, such as resveratrol (RES), have been shown to have significant anti-aging effects on yeast and positive effects on AD via the SIRT1 signaling pathway, a pathway that’s known to have important functions in aging and AD [1–7]. Since no effective treatment drugs are available for AD, studies on anti-aging substances have great significance in AD treatment.

**Figure 1:**
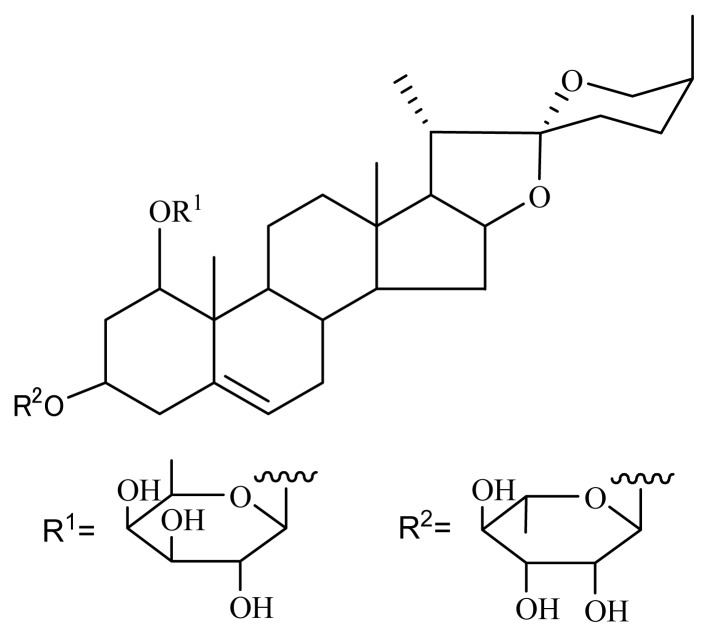
Chemical structure of nolinospiroside F.

One of the best-known methods used in anti-aging studies is the yeast replicative lifespan assay. A new bioassay method was established with the K6001 yeast strain, which is more convenient than the traditional lifespan assay [8]. By using the new assay, we screened several anti-aging compounds, such as ganodermasides A–D, phloridzin and hesperidin [9–12]. In the present study, we have isolated a known steroidal saponin, nolinospiroside F, from *O. japonicus* (maidong in Chinese) (Figure 1) [13].

Steroidal saponins are molecules with various bioactivities. Steroidal saponins are mainly found in plants and some marine organisms, such as starfish [14–16]. Previous studies have shown that steroidal saponins have anti-AD, anti-tumor, anti-inflammatory, anti-thrombotic and anti-fungal activities [17–21]. Because of their diverse biological activities, steroidal saponins are extensively studied and have attracted commercial interest. However, few studies on the anti-aging effect of steroidal saponins have been reported. Although nolinospiroside F was isolated in 1983 [22], very few reports on its biological activity have been published. In the present paper, we report that nolinospiroside F possesses anti-aging effects on the yeast replicative lifespan by regulating each other’s expression of *SOD* and *UTH1* genes.

## 2. Results and Discussion

### 2.1. Nolinospiroside F Has Anti-Aging Effects in Yeast

**Figure 2:**
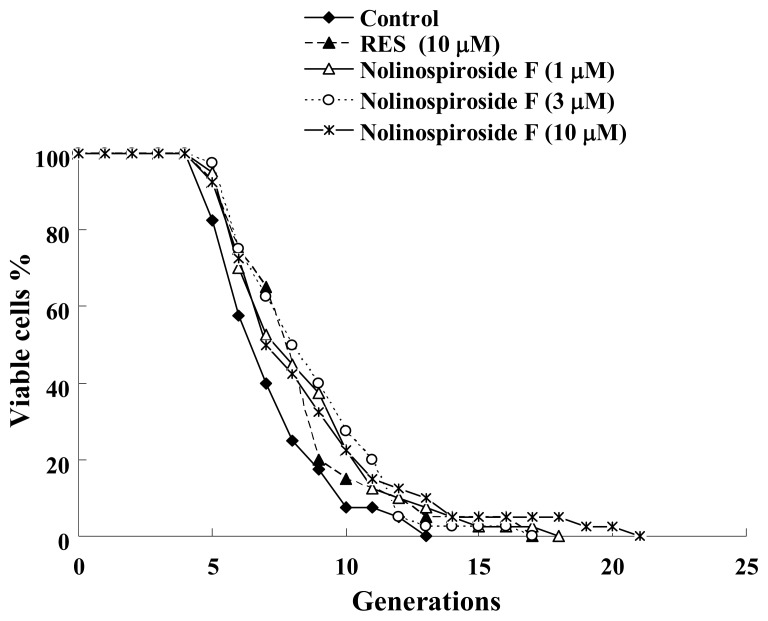
Effects of nolinospiroside F on the replicative lifespan of K6001 yeast strain. The yeast cells grown in YPGal medium were spread on a glucose medium plate containing different nolinospiroside F concentrations. The daughter cells of 40 microcolonies of each experiment were counted. The assay was repeated at least thrice. The average lifespan of untreated K6001 was 6.43 generations; resveratrol (RES) at 10 μM, 7.55*; nolinospiroside F at 1 μM, 7.65*; at 3 μM, 7.88**; and at 10 μM, 7.80* (* *p* < 0.05, ** *p* < 0.01).

When screening natural products, the bioassay used must be simple, reliable and fast and have good reproducibility. The K6001 yeast strain meets these requirements, as proven in studies with a well-known small molecule, resveratrol (RES) [8]. In previous studies, anti-aging compounds were screened by using this bioassay, which included novel ganodermasides A–D isolated using this bioassay from the spores of the medicinal mushroom, *Ganoderma lucidum*, and phloridzin extracted from apple branch [9–11]. In this study, the bioassay was used to investigate the anti-aging effects of nolinospiroside F. The results show that nolinospiroside F extends the yeast replicative lifespan significantly at concentrations of 1, 3 and 10 μM (*p* < 0.05, *p* < 0.01 and *p* < 0.05, respectively) (Figure 2), indicating that it has anti-aging effects in yeast.

### 2.2. Nolinospiroside F Affects the Growth of Yeast under Oxidative Stress Conditions

**Figure 3:**
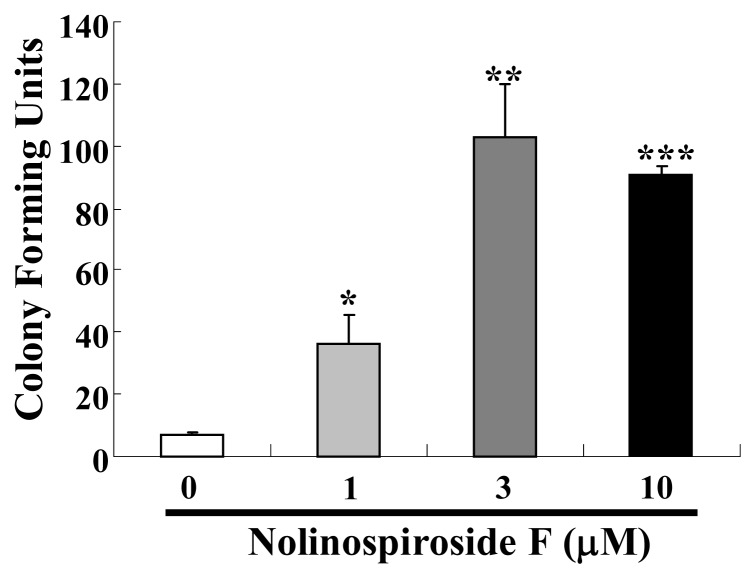
Protection effect of nolinospiroside F on the growth of K6001 yeast under oxidative stress conditions. K6001 yeast was incubated for 36 h after treatment with 1, 3 or 10 μM nolinospiroside F. Then, 5 μL aliquots with the same number of cells were spotted onto plates containing 7.5 mM H_2_O_2_. The plates were incubated for 3 d at 28 °C, and the number of colonies was counted. The assay was repeated at least thrice. Vertical bars represent the mean ± S.E.M. of three assays. The average number of colonies of untreated K6001 was seven; nolinospiroside F at 1 μM, 37*; at 3 μM, 103**; and at 10 μM, 91*** (* *p* < 0.05, ** *p* < 0.01, *** *p* < 0.001).

Among the various theories used to explain the aging process, the activity of free radicals, which was first proposed by Harman [23], has received great attention. The principle behind this theory is that the accumulation of reactive oxygen species (ROS), the by-products of aerobic activities in living organisms, shortens their lifespan [24]. The damage brought about by ROS contributes to aging. Thus, we investigated whether or not nolinospiroside F affects anti-oxidative stress. Under oxidative conditions (treatment with 7.5 mM H_2_O_2_), the yeast growth was significantly improved by nolinospiroside F at concentrations of 1, 3 and 10 μM (*p* < 0.05, *p* < 0.01 and *p* < 0.001, respectively) (Figure 3). These results suggest that nolinospiroside F may have an anti-oxidative effect, which may contribute to its anti-aging effect.

### 2.3. Nolinospiroside F Decreases MDA Levels

**Figure 4:**
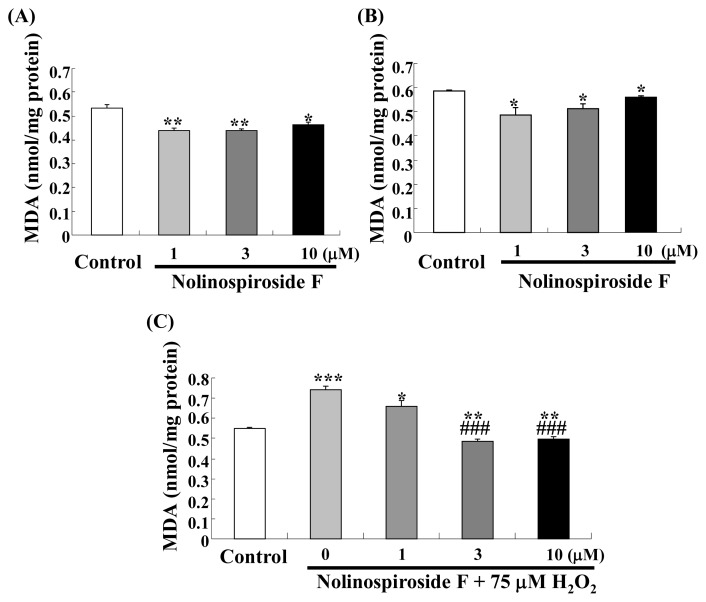
Change in malondialdehyde (MDA) level in yeast after nolinospiroside F treatment at 24 h and 48 h (**A**, **B**) under normal and oxidative stress condition (**C**). K6001 yeast cells were incubated with various nolinospiroside F concentrations for 24 h and 48 h. In oxidative stress experiment, K6001 yeast cells were precultured with nolinospiroside F for 12 h, then 75 μM H_2_O_2_ was added and continuously incubated for 24 h. Changes in MDA level were measured with an MDA assay kit according to the protocol. Values shown are the mean ± S.E.M. of three independent experiments. *, ** and *** indicate significant difference relative to the control (*p* < 0.05, *p* < 0.01, *p* < 0.001); ^###^ represents obvious difference among the group treated with 75 μM H_2_O_2_ (*p* < 0.001).

Malondialdehyde (MDA), the principal product of the degradation of polyunsaturated lipids by ROS [25], is an oxidative stress biomarker in organisms. MDA can cause membrane damage, add fluidity to cells and damage DNA [26–28]. Because nolinospiroside F has anti-oxidative effects, we determined the change in MDA after treatment with the steroid saponin in normal and oxidative stress condition. MDA levels decreased significantly after treatment with 1, 3 and 10 μM nolinospiroside F for 24 h (*p* < 0.01, *p* < 0.01, *p* < 0.05) (Figure 4A) and 48 h (*p* < 0.05, *p* < 0.05, *p* < 0.05) (Figure 4B). At the same time, the inhibition effects of nolinospiroside F on MDA under oxidative stress condition led by 75 μM H_2_O_2_ were observed (*p* < 0.001, *p* < 0.001) (Figure 4C). These results suggested that nolinospiroside F may have protective effects for cell membrane and DNA via inhibition of MDA level.

### 2.4. Treatment with Nolinospiroside F Increases *SOD* Gene Expression and SOD Enzyme Activity

**Figure 5:**
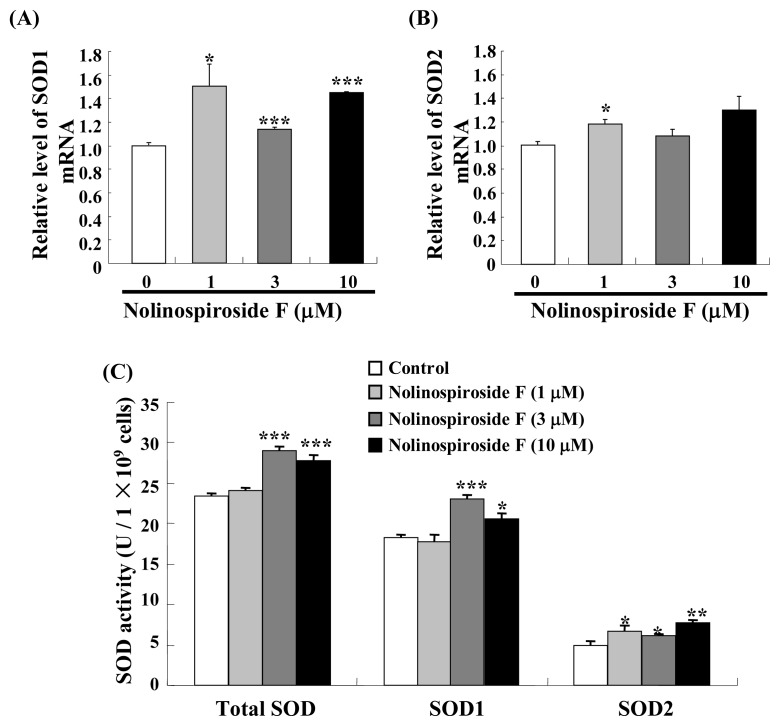
Effects of nolinospiroside F on *SOD* gene expression (**A**, **B**) and enzyme activity (**C**). Amplification of TUB1 was used to normalize the relative levels of *SOD1* and *SOD2* mRNA. A superoxide dismutase (SOD) activity assay kit was used to measure SOD activity. Vertical bars represent the mean ± S.E.M. of three assays. Asterisks indicate significant differences compared with the corresponding controls (*p < 0.05, **p < 0.01, ***p < 0.001).

*SOD* is an important anti-oxidative stress gene that participates in free radical scavenging. We hypothesized that *SOD* may participate in the anti-aging effects of nolinospiroside F. Thus, we evaluated *SOD* gene expression and superoxide dismutase (SOD) activity in yeast treated with nolinospiroside F. *SOD1* expression was significantly increased in the treatment groups compared with the untreated group after treatment with 1, 3 and 10 μM nolinospiroside F (*p* < 0.05, *p* < 0.001 and *p* < 0.001, respectively) (Figure 5A). By contrast, only 1 μM nolinospiroside F resulted in a significant increase in *SOD2* mRNA (*p* < 0.05) (Figure 5B). The total SOD (*p* < 0.001, *p* < 0.001) and SOD1 (*p* < 0.001, *p* < 0.05) activity significantly increased after yeast was treated with 3 and 10 μM nolinospiroside F (Figure 5C) for 48 h, whereas SOD2 activity increased significantly after treatment with 1, 3 and 10 μM nolinospiroside F (*p* < 0.05, *p* < 0.05 and *p* < 0.01, respectively) (Figure 5C). These results suggest that nolinospiroside F maybe extend the yeast lifespan and increase antioxidant defenses by increasing *SOD* gene expression and enzyme activity.

During *SOD1* and *SOD2* expression analysis, *SOD1* expression increased significantly after treatment with 1, 3 and 10 μM nolinospiroside F. By contrast, *SOD2* expression increased only with 1 μM nolinospiroside F. *SOD1* located in the cytoplasm is believed to be very stable, whereas mitochondrial *SOD2* is highly regulated by a number of environmental factors. However, our results are not consistent with the previous study; the *SOD2* gene is more sensitive than *SOD1* for hesperidin [12]. The difference between *SOD1* and *SOD2* expression may be due to the fact that *SOD1* expression is easily triggered by saponins, as previously reported [29]. Total SOD and SOD1 enzyme activity did not change after treatment with 1 μM nolinospiroside F for 48 h. We determined changes in them 24, 48 and 72 h after treatment with 1 μM nolinospiroside F and found that total SOD and SOD1 activities increased 24 h after administration of 1 μM nolinospiroside F, but not 48 or 72 h later (data not shown). This result indicates that nolinospiroside F could be consumed by the yeast after long-term incubation.

### 2.5. Nolinospiroside F Does Not Affect the Lifespans of *sod1* and *sod2* Yeast Mutant Strains

**Figure 6:**
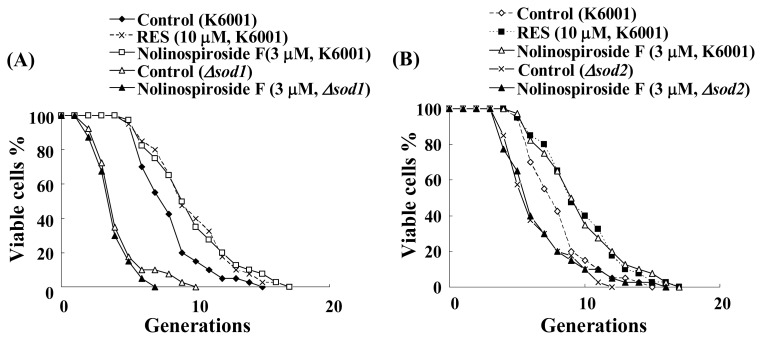
Effects of nolinospiroside F on the replicative lifespan of *sod1* (**A**) and *sod2* (**B**) mutant yeast strains with a K6001 background. The assay is described in Figure 2. The daughter cells of 40 microcolonies of each experiment were counted. The assay was repeated at least thrice. The average lifespan of untreated K6001 was 6.35 generations; RES at 10 μM, 8.83***; nolinospiroside F at 3 μM, 8.75***; *sod1* mutant with a K6001 background was 3.50; nolinospiroside F at 3 μM, 2.83; *sod2* mutant with a K6001 background was 5.60; nolinospiroside F at 3 μM, 5.80. *Δsod1* and *Δsod2* represent the *sod1* and *sod2* mutants yeast strain with a K6001 background, respectively. (*** *p* < 0.001).

To confirm whether or not *SOD* participates in the anti-aging effect of nolinospiroside F, we used *sod1* and *sod2* mutant yeast strains to examine the effects of nolinospiroside F effects on replicative lifespan. Shorter lifespans of *sod1* and *sod2* mutant yeast strains with the K6001 background were observed in our experiment, and nolinospiroside F did not affect the lifespans of *sod1* and *sod2* mutant yeast strains (Figure 6). These results indicate that *SOD* plays an important role in the anti-aging effect of nolinospiroside F.

### 2.6. Nolinospiroside F Increased SIRT1 Activity

**Figure 7:**
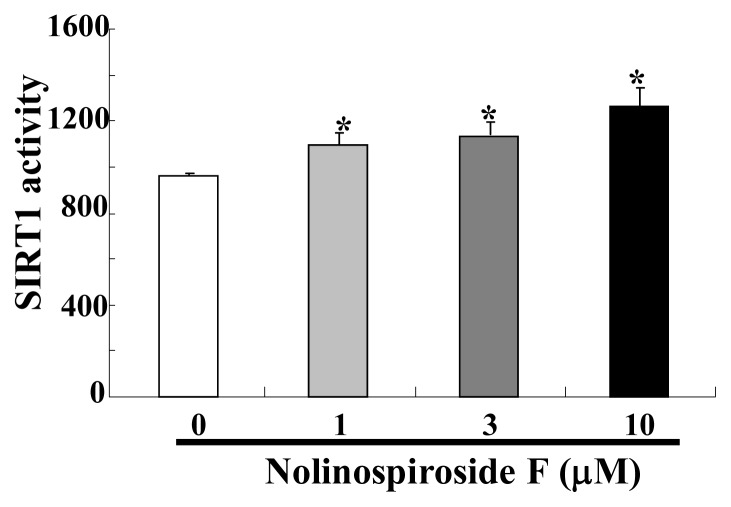
Effects of nolinospiroside F on SIRT1 activity. Values shown are the mean ± S.E.M. of three independent experiments. (*p < 0.05).

SIRT1, the homolog of yeast Sir2, has an important function in the lifespan of mammals. SIRT1 can deacetylate and activate PGC-1α and FOXOs to induce *SOD2* expression [30,31]. It can also increase *SOD2* expression to promote cell survival [32]. Thus, the effect of nolinospiroside F on SIRT1 activity was investigated. By using a SIRT1/Sir2 activity kit, we found SIRT1 activity increased obviously after treatment with 1, 3 and 10 μM nolinospiroside F (*p* < 0.05, *p* < 0.05, *p* < 0.05) (Figure 7). This result implied that Sir2 protein may be involved in the anti-aging effects of nolinospiroside F. To further confirm whether Sir2 participates in the anti-aging effect of nolinospiroside F, we tried to construct *sir2* mutation yeast with K6001 background and did a replicative lifespan assay. However, the mother cells of K6001 yeast did not reproduce daughter cells after deleting the *Sir2* gene. This result was consistent with other report [8]. Therefore, we could not utilize the lifespan assay of *sir2* mutation yeast with a K6001 background to test the effects of nolinospiroside F on the *Sir2* gene in this study.

### 2.7. Nolinospiroside F Administration Inhibits *UTH1* Gene Expression and *UTH1, SKN7* Are Involved in Nolinospiroside F-Mediated Lifespan Extension

**Figure 8:**
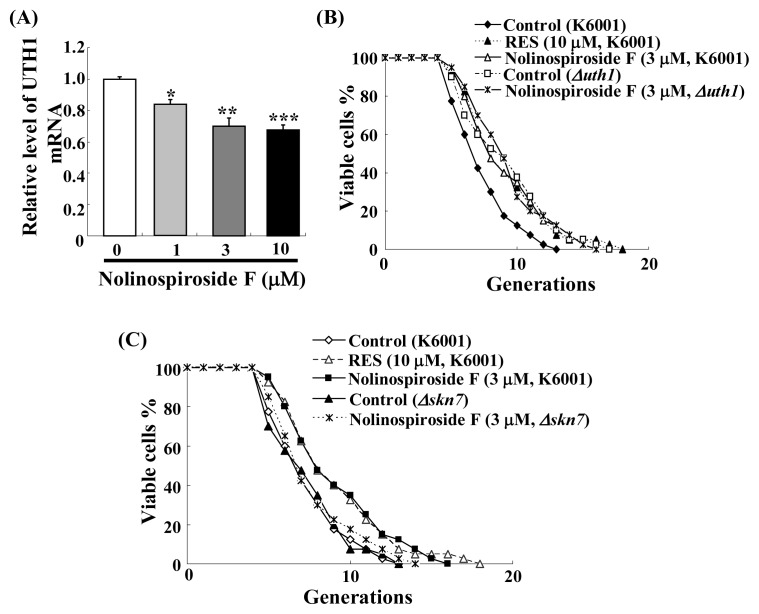
Inhibition of *UTH1* gene expression by nolinospiroside F (**A**) and effects of nolinospiroside F on the replicative lifespan of *uth1* (**B**) and *skn7* (**C**) mutants of K6001 yeast. Daughter cells of 40 microcolonies of each experiment were randomly counted. The assay was repeated at least thrice. The average lifespan of untreated K6001 was 6.50; RES at 10 μM, 8.20**; nolinospiroside F at 3 μM, 8.40**; *uth1* mutant with a K6001 background was 8.20; nolinospiroside F at 3 μM, 8.50; untreated *skn7* mutant with a K6001 background was 6.50 generations; nolinospiroside F at 3 μM, 6.88. Δ*uth1* and Δ*skn7* represents *uth1* and *skn7* mutant yeast strains with a K6001 background. (*p < 0.05, **p < 0.01, ***p < 0.001).

*UTH1* is a yeast-aging gene that is reportedly involved in the oxidative stress of yeast [33]. Deletion of *UTH1* increases the yeast lifespan [34,35]. Thus, we hypothesized that the *UTH1* gene may be involved in nolinospiroside F-mediated yeast lifespan extension. Real-time polymerase chain reaction (RT-PCR) analysis and a lifespan assay of *uth1* mutant were performed. *UTH1* gene expression was inhibited significantly in groups treated with 1, 3 and 10 μM nolinospiroside F (*p* < 0.05, *p* < 0.01 and *p* < 0.001, respectively) (Figure 8A). The lifespan of the *uth1* mutant was found to be longer than that of the K6001 yeast strain, which is consistent with other reports [34,35]. Nolinospiroside F did not affect the lifespan of *uth1* mutants at a concentration of 3 μM (Figure 8B). These results suggest that *UTH1* has an important role in nolinospiroside F-mediated yeast lifespan extension.

SKN7 is a transcriptional activator. In previous studies, we found that *skn7* mutant yeast represses *UTH1* expression significantly [12]. To observe whether or not SKN7 is important for the lifespan extension effect by nolinospiroside F, a lifespan assay of *skn7* mutant yeast with a K6001 background was performed. Nolinospiroside F did not affect the lifespan of *skn7* or *uth1* mutant at 3 μM (Figure 8C), which indicates that SKN7 exerts effects on nolinospiroside F-mediated yeast lifespan extension by inhibiting *UTH1* gene expression.

### 2.8. The Relationship between the *SOD* and *UTH1* Gene

**Figure 9:**
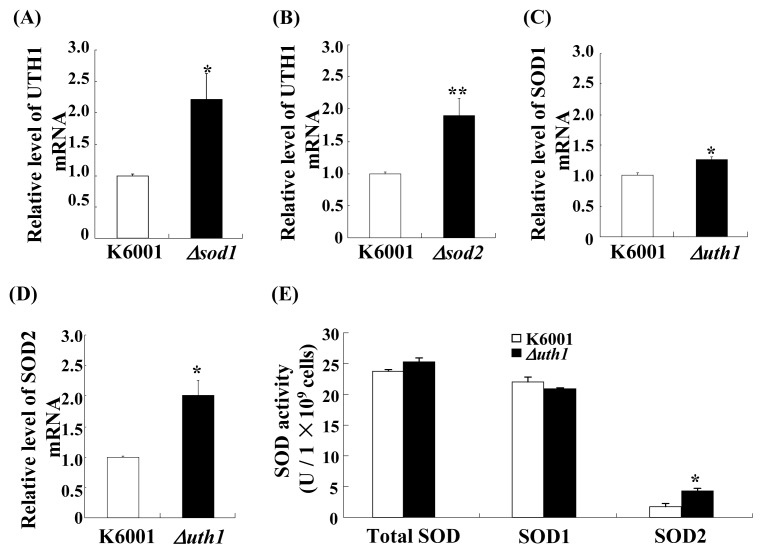
The relationship between *SOD* and *UTH1. UTH1* expression of *sod1* and *sod2* mutant yeast strains with a K6001 background was investigated (**A** and **B**). *SOD1*, *SOD2* expressions and SOD enzyme activity of the *uth1* mutant yeast strain with K6001 background were determined (**C**, **D** and **E**). *UTH1* increased significantly when the *SOD1* or *SOD2* gene was deleted. The *SOD1*, *SOD2* expressions and SOD2 enzyme activity of *uth1* mutants were increased significantly, compared with the K6001 yeast strain. Values shown are the mean ± S.E.M. of three independent experiments (*p < 0.05, **p < 0.01).

In this study, both *UTH1* and *SOD* were found to be important for nolinospiroside F-mediated yeast lifespan extension, and these genes may have other important functions in yeast oxidative stress. *UTH1* is reported to have a close relationship with *SOD*[36]. To explore this relationship further, *SOD1* and *SOD2* expressions in a *uth1* yeast mutant strain and *UTH1* expression in *sod1* and *sod2* mutants were investigated. The *UTH1* expression in *sod1* and *sod2* mutants increased significantly compared to that in K6001 yeast (*p* < 0.05, *p* < 0.01, respectively); the *SOD1* and *SOD2* expressions in the *uth1* mutant yeast strain also increased significantly (*p* < 0.05, *p* < 0.05) (Figure 9A–D). Furthermore, the significant increase of SOD2 activity in the *uth1* mutant was observed (*p* < 0.05) (Figure 9E). These results suggest that *UTH1* and *SOD* regulate expression with each other in yeast cells.

## 3. Materials and Methods

### 3.1. Isolation of Nolinospiroside F

*O. japonicus* was bought in Zhejiang Province, China. Dried *O. japonicus* (dry wt: 10 kg) was extracted with MeOH (50 L). The supernatant was separated by filtration and concentrated to obtain 1.5 kg of extracts. The extract was then partitioned between H_2_O and EtOAc. The EtOAc layer was concentrated to obtain 8.3 g of the dried sample. Part of the EtOAc layer (0.73 g) was chromatographed on silica gel (200 to 300 mesh, Yantai Chemical Industry Research Institute) using CHCl_3_/MeOH (10:0, 9:1, 8:2, 5:5 and 0:10) to give five parts. The active sample eluted with CHCl_3_/MeOH (8:2 and 5:5) was separated through octadecylsilane (ODS) (Cosmosil 75 C_18_-OPN, Nacalai Tesque) and successively eluted with MeOH/H_2_O (4:6, 5:5, 7:3, 8:2, 9:1 and 10:0) and MeOH/CHCl_3_ (5:5) to obtain seven parts. The active sample (64.3 mg total) eluted with MeOH/H_2_O (7:3) was subjected to high-performance liquid chromatography (Develosil ODS-HG-5 (20/250 mm), Nomura Chemicals, flow rate: 8 mL/min, MeCN/H_2_O (5:5)) to yield the pure compound nolinospiroside F (21.5 mg, *t*_R_ = 50 min). The chemical structure of the compound was determined by comparing ^1^H and ^13^C nuclear magnetic resonance (NMR), mass spectrometry (MS), and optical rotation data with those reported in the literatures [13,22]. The ^13^C NMR chemical shifts in ppm were referenced to the solvent peak of δc 135.91 for pyridine-*d_5_*. δ 14.98, 15.20, 16.66, 17.19, 17.78, 18.98, 24.20, 26.57, 26.74, 27.91, 32.34, 32.78, 33.38, 36.30, 39.72, 40.56, 40.75, 42.83, 43.32, 50.93, 57.35, 63.30, 65.40, 70.48, 71.50, 72.54, 72.87, 73.18, 73.18, 73.85, 74.51, 75.68, 81.58, 84.21, 100.31, 103.01, 110.11, 126.07 and 138.66; MS (*m*/*z* 745 (M + Na)^+^); [α]_D_^25^= −108.5 (*c* 0.21, pyridine).

### 3.2. *Saccharomyces cerevisiae* Strains, Media and Lifespan Assay

Strains were derived from K6001 yeast. *Sod1*, *sod2*, *uth1* and *skn7* mutants with a K6001 background were constructed using the kanamycin gene instead of the target gene. The yeast lifespan assay and media were described in a previous report [12]. Briefly, K6001 yeast strain was inoculated in 2 mL of galactose medium and incubated in a shaking incubator at 28 °C and 160 rpm for 48 h. Yeast culture was centrifuged at 1500 rpm for 3 min. After that, the yeast pellet was washed thrice and diluted with distilled water. Subsequently, yeast were counted with a hemocytometer, 5000 cells were plated on glucose medium agar plates containing various concentrations of the samples and plates were incubated at 28 °C for two days. Microcolonies that formed on agar plates were observed under a microscope, and daughter cells were counted.

### 3.3. Anti-Oxidative Stress Assay

After treatment with 0, 1, 3 or 10 μM nolinospiroside F, the K6001 yeast cells were cultured in 25 mL of galactose medium for 36 h. About 5 μL of the same OD600 aliquot of cultured yeast was spotted onto agar plates containing 7.5 mM H_2_O_2_. Incubation at 28 °C followed. After 3 d, the number of yeast colonies was counted.

### 3.4. MDA Assay

K6001 yeast cells were cultured in galactose medium after treatment with 0, 1, 3 or 10 μM nolinospiroside F for 24 or 48 h. To measure the MDA change in cells after administrating nolinospiroside F under oxidative stress condition, K6001 yeast cells were pretreated with 0, 1, 3 or 10 μM nolinospiroside F for 12 h. After that, 75 μM H_2_O_2_ was added into the cell culture, and yeast cells were incubated continuously for 24 h. The cells were washed thrice with PBS and collected after centrifugation at 3000 rpm for 10 min. Cells were resuspended in 200 μL of PBS and disintegrated by ultrasonication (1 min/time, 5 times) and freeze-thawing (5 min in liquid nitrogen, then 15 min in water bath at 37 °C) 5 times, followed by repeated ultrasonication 5 times. The cell lysate was centrifuged at 12,000 rpm at 4 °C for 15 min, after which the supernatant was removed. MDA was measured using an MDA assay kit following the manufacturer’s instructions (Nanjing Jiancheng Bioengineering Institute, Nanjing, China).

### 3.5. RT-PCR Analysis

RT-PCR was performed as previously described [12]. Primers used were as follows: for *SOD1*, sense 5′-CAC CAT TTT CGT CCG TCT TT-3′ and antisense 5′-TGG TTG TGT CTC TGC TGG TC-3′; for *SOD2*, sense 5′-CTC CGG TCA AAT CAA CGA AT-3′ and antisense 5′-CCT TGG CCA GAA GAT CTG AG-3′; for *UTH1*, sense 5′-CGC CTC TTC TTC CTC CTC TT-3′ and antisense 5′-ACC ATC GGA AGG TTG TTC AG-3′; for *TUB1*, sense 5′-CCA AGG GCT ATT TAC GTG GA-3′ and antisense 5′-GGT GTA ATG GCC TCT TGC AT-3′. The amounts of *UTH1*, *SOD1* and *SOD2* mRNA were normalized to that of *TUB1.*

### 3.6. *SOD* Activity Assay

K6001 yeast cells were cultured in galactose medium after treatment with 0, 1, 3 or 10 μM nolinospiroside F for 48 h. K6001 or Δ*uth1* with K6001 background yeast cells were cultured in galactose medium for 48 h, respectively. Approximately 1 × 10^9^ cells were washed thrice with PBS and resuspended in 1 mL of PBS. Cells were frozen and thawed (5 min in liquid nitrogen, then 15 min in water bath at 37 °C) thrice. Cell lysates were centrifuged at 10,000 rpm and 4 °C for 15 min, after which the supernatant was removed. Finally, SOD activity was measured using a SOD assay kit, according to the manufacturer’s instructions (Nanjing Jiancheng Bioengineering Institute, Nanjing, China).

### 3.7. SIRT1 Assay

Different concentrations of nolinospiroside F were added to the reagents of an SIRT1/Sir2 assay kit, according to the manufacturer’s instructions (CycLex, Ina, Nagano, Japan). The detailed procedure for the SIRT1 assay was described in a previous report [12].

### 3.8. Statistical Analysis

Significant differences among groups in all experiments were determined by ANOVA, followed by two-tailed multiple *t*-tests with Bonferroni correction using SPSS biostatistics software. A *p*-value less than 0.05 was considered statistically significant.

## 4. Conclusion

In this study, we found that nolinospiroside F extends the replicative lifespan of the K6001 yeast strain significantly and improves yeast survival under oxidation conditions. It also increases *SOD1* and *SOD2* expressions, SOD enzyme activity and SIRT1 activity. MDA levels and *UTH1* expression decreased after nolinospiroside F treatment. We also confirmed the function of *SOD* and *UTH1* in the yeast lifespan extension effects of nolinospiroside F using a replicative lifespan assay with *sod1*, *sod2* and *uth1* mutant strains. *SOD* and *UTH1* regulated expression with each other. The results suggest that the anti-aging effects of nolinospiroside F are a result of the multimodulation of mitochondrial functions via *SOD* and *UTH1*. Furthermore, the anti-aging effect of nolinospiroside F should be investigated *in vivo* in future experiments.
